# On the Use of Focused Incident Near-Field Beams in Microwave Imaging

**DOI:** 10.3390/s18093127

**Published:** 2018-09-17

**Authors:** Nozhan Bayat, Puyan Mojabi

**Affiliations:** Department of Electrical and Computer Engineering, University of Manitoba, Winnipeg, MB R3T 5V6, Canada; Puyan.Mojabi@umanitoba.ca

**Keywords:** microwave imaging (MWI), near-field (NF) zone, sub-wavelength focusing, near-field plates, Bessel beams, inverse scattering, inverse source problems

## Abstract

We consider the use of focused incident near-field (NF) beams to interrogate the object of interest (OI) in NF microwave imaging (MWI). To this end, we first discuss how focused NF beams can be advantageously utilized to suppress scattering effects from the neighbouring objects whose unknown dielectric properties are not of interest (i.e., undesired scatterers). We then discuss how this approach can also be helpful in reducing the required measured data points to perform imaging. Driven by the relation between the electromagnetic inverse source and inverse scattering problems, our approach emphasizes the importance of tailoring the induced contrast sources in the imaging domain through the utilized incident NF beams. To demonstrate this idea, we consider two recently-proposed NF beams, and simulate them for imaging applications. The first one is a subwavelength focused NF beam generated by a passive NF plate, and the other is a Bessel beam generated by a leaky radial waveguide. Simple imaging examples are considered to explore the potential advantages of this approach, in particular, toward mainly *seeing* the object of interest, and not the unknown undesired scatterers. The scope of this paper is limited to homogeneous dielectric objects for which the induced total field distributions in the interrogated objects are similar to the incident field distributions (e.g., those that satisfy the Born approximation). Simple inversion results for focused and non-focused beams are presented accompanied by discussions comparing the achieved reconstructed values.

## 1. Introduction

Microwave imaging (MWI) is an imaging technique that can be used to produce a quantitative image of the dielectric profile of the object of interest (OI) by solving the corresponding electromagnetic inverse problem. In MWI, the OI is illuminated by incident electromagnetic fields at the microwave frequency range, and the resulting scattered fields are processed (inverted) to create (reconstruct) the OI’s dielectric profile image. This processing often involves calibrating the measured data and applying an appropriate inversion algorithm to this calibrated measured data. This imaging tool has the potential to be utilized for different applications such as breast cancer detection, stroke diagnosis, through wall imaging, security screening, and industrial non-destructive evaluation [1,2,3,4,5,6,7,8,9,10]. MWI can also be used in conjunction with other imaging tools, e.g., with magnetic resonance imaging or ultrasound tomography [11,12,13]. In addition, MWI can be performed in the time domain or frequency domain, with the latter being the focus of this paper. Moreover, in some applications, reconstructing the magnetic properties is also of interest (e.g., in [14]); herein, we only consider non-magnetic objects.

MWI can be performed in at least three fashions: (i) 1D (line reconstruction; e.g., [15]); (ii) 2D (cross section reconstruction; e.g., [16]), and (iii) 3D (volumetric reconstruction; e.g., [17]). This is often determined based on several factors such as the application area, data collection process, and the amount of measured data. For some applications including biomedical imaging, it is desirable to enhance the achievable reconstruction accuracy from MWI. To this end, in addition to the development of appropriate inversion algorithms and regularization techniques, e.g., [18,19,20,21], several other techniques have been suggested to further improve the achievable image accuracy from MWI. For example, these include (i) using prior spatial information about the OI [22,23,24], (ii) using prior information about the permittivity values or the expected ratio between the real and imaginary parts of the OI’s complex permittivity [25,26]; (iii) increasing the number of transceivers and frequencies of operation [27]; (iv) improving the signal to noise ratio (SNR) of the system [28]; (v) appropriate data calibration techniques [7,29]; (vi) enhanced modeling of the imaging system in the inversion algorithm [30]. All of the above techniques may be classified under the following two categories. The first category aims to increase the *overall* SNR with noise being either the actual noise or the modeling error. (Modeling error is defined as any discrepancies between the actual imaging setup and the numerical model used in the inversion algorithm.) On the other hand, the second category aims to enrich the information content of the data to be used for inversion; this is done by increasing the number of measurements and incorporating prior information (virtual data) about the OI.

The work presented in this paper aims to suppress the effect of *undesired* scatterers in the inversion process. To this end, we begin by defining undesired scatterers as any scatterers within the imaging chamber in which we are *not* interested in finding their *unknown* dielectric properties. Considering the scattered fields due to these undesired scatterers as unwanted signals, or *noise*, the topic of this paper, therefore, falls under the first category above: i.e., enhancing the overall SNR. Note that the undesired scatterer and the OI might be two distinct objects, or they can be attached to each other. For example, in 2D (cross-sectional) imaging of a 3D object, e.g., breast, the irradiating antenna not only illuminates the cross-section of interest but also illuminates other cross-sections. These other cross sections are then undesired scatterers with respect to the cross section to be imaged. Herein, we investigate how tailoring the incident field can be helpful toward suppressing such undesired scattering effects. Since the focus of this paper is on the incident field (and not the total field), the scope of this work is limited to imaging scenarios in which the induced fields in the dielectric objects are similar to the incident fields. (This is mainly associated with low-contrast and electrically small objects.) In addition, for simplicity, we have also limited the scope of this work to homogeneous lossless OIs and undesired scatterers.

A key aspect which is considered in this paper is that the OI is often placed electrically close to the antenna system, i.e., in its near-field (NF) zone, so as to make the imaging system more compact and also to enrich the information content of the measured data. (NF data collection increases the chance of capturing evanescent waves that contain high spatial resolution information about the OI.) Several NF MWI systems have been developed; however, to the best of our knowledge, all of them utilize standard “far-field (FF) antennas”. Herein, the term “FF antennas” has been used to indicate standard antennas such as dipoles, monopoles, Vivaldi, open ended waveguides which have not been specifically designed to achieve a certain NF distribution. (Some of these FF antennas have been modified, e.g., with a dielectric inclusion, to enhance their NF focusing [31,32].) All of these FF antennas have important advantages, e.g., their compactness, ease of modelling in the inversion algorithm, bandwidth, and the ability to easily operate in matching fluids. However, it is beneficial for near-field MWI to investigate antennas which are specifically designed to achieve desired NF distributions: in NF MWI, it is the incident NF of the antenna, *not* its FF pattern, that interrogates the OI. As a follow-up to our previous work where we have discussed that the choice of the incident field distribution can affect the achievable reconstruction [27,33], we now discuss and demonstrate that a focused incident NF can be used advantageously in NF MWI to suppress undesired scattering events. The idea behind this paper is therefore simple: making sure that the *spotlight* of the antenna is on the OI, and not on the undesired scatterers. To this end, we will utilize a NF plate and a Bessel beam launcher to demonstrate the potential advantages of using a focused incident NF beam in MWI. (We have recently presented a concise form of this idea in [34].) We also note that there exist other techniques for focusing the fields into a hotspot, e.g., using an antenna array configuration [35,36,37]. The focus of this paper is not to compare these focusing techniques; we just note that the radiators used in this paper do not require array feeding networks or array signal processing.

Finally, in this paper, we use the terms *focused* instead of *directive*, and *distribution* instead of *pattern* since the terms *directivity* and *pattern* describe FF properties of antennas, and are more appropriate for the FF zone. We also utilize *NF distributions* and *NF beams* interchangeably. In addition, hereafter, we refer to NF MWI simply as MWI for brevity. It should also be noted that the time-dependency of exp(jωt) is implicitly assumed throughout this paper.

## 2. Motivation

Incorporating unknown undesired scatterers in the inversion process requires that we (i) include them as extra unknowns in the inversion algorithm, and subsequently (ii) ensure that more scattering data are collected to compensate for the resulting increase in the number of unknowns. However, we may not be able to meet these two conditions in certain cases. To understand this better, it is important to discuss the “sufficient” amount of data needed for successful inversion. To this end, we begin by reviewing how the MWI problem, which is an inverse scattering problem, is related to the inverse source problem. (In the inverse source problem, the goal is to find the equivalent currents that generate the measured electromagnetic fields.) Once this relation is established, the amount of information needed for successful inversion can be better understood from NF antenna measurements’ point of view. We then address how the choice of the NF distribution can be utilized advantageously to suppress the scattering from undesired scatterers in an attempt to alleviate the necessity of including them as extra unknowns in the inversion process.

### 2.1. The Relation between the MWI and Inverse Source Problems

**Figure 1:**
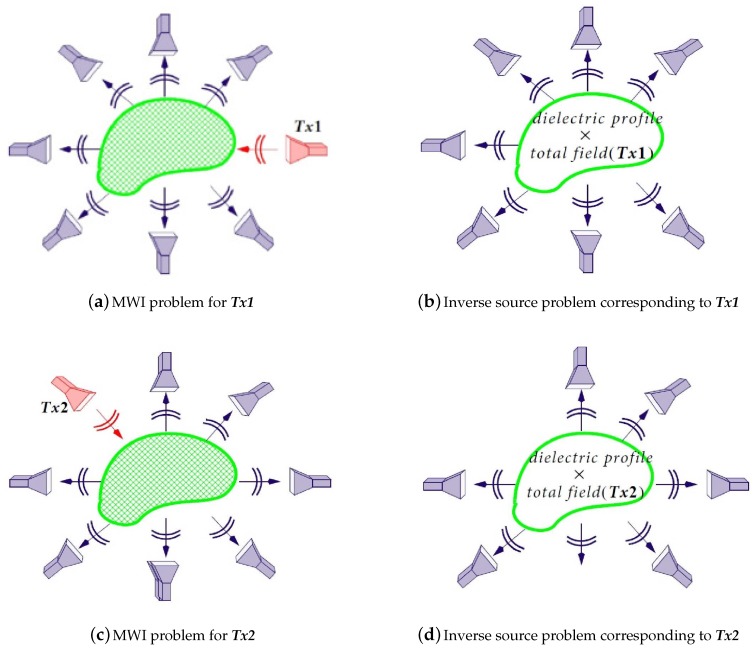
Demonstration of the relation between the MWI and inverse source problems. (**a**) represents the MWI problem when the OI is interrogated by the transmitter ***Tx1*** (red antenna) while the other antennas (grey antennas) act as receivers; (**b**) represents the equivalent inverse source problem for (**a**) in which ***Tx1*** has been replaced by contrast sources in the geometrical domain of the OI; similarly (**c**,**d**) demonstrate the same concept but for a different transmitter: ***Tx2***.

Consider Figure 1a in which the dashed green region represents the OI surrounded by multiple antennas. As can be seen, the antenna denoted by Tx1 irradiates the OI. The other antennas surrounding the OI act as receivers, and collect the resulting *total* field data E. The incident field $E^{\mathrm{inc}}$, i.e., the field in the absence of the OI, is also collected in a separate experiment by the same antennas. Having both the total and incident fields, the scattered field can then be obtained as $E^{\mathrm{scat}}≜E-E^{\mathrm{inc}}$. Equivalently, this scattered field can be thought as the field radiated by a new set of currents, often referred to as *contrast sources* [38] which radiate in the background medium. These contrast sources are confined within the OI’s geometrical domain; i.e., they are zero outside the OI’s geometrical support. This is shown in Figure 1b where the actual illuminating antenna Tx1 is replaced by contrast sources within the OI’s geometrical domain. As shown in this figure, these contrast sources depend on the multiplication of the OI’s dielectric contrast profile and the induced total field within the OI due to Tx1. These contrast sources are then written as w(r)=χ(r)E(r) [38] where χ is the dielectric contrast profile of the OI, E is the total field induced in the OI due to a given transmitter (in this case, Tx1), and r is the location vector. Note that the dielectric contrast profile is defined as

(1) $$\chi \left(r\right)≜\frac{\epsilon \left(r\right)-\epsilon_{b}}{\epsilon_{b}},$$

where ϵ(r) is the relative permittivity of the OI, and $\epsilon_{b}$ is the relative homogeneous permittivity of the background medium. (In this paper, the background medium is air.) As can be seen from the above discussion, the MWI problem in Figure 1a has now been cast as an electromagnetic inverse source problem shown in Figure 1b, with its unknown quantity being these contrast sources. (This well-known relation can also be found in other references, e.g., see [39].) To minimize the null space of the associated inverse problem, the OI may be illuminated from multiple angles. This has been demonstrated in Figure 1c in which a different transmitter (Tx2) now illuminates the OI. Similarly, this is equivalent to the inverse source problem depicted in Figure 1d; however, the new unknown contrast sources in this case are different than the ones in Figure 1b as the total field induced in the OI is now due to Tx2, instead of Tx1. Based on this discussion, the MWI problem can be thought as the summation of multiple inverse source problems whose unknown contrast sources share one common component: the dielectric profile of the OI. For a more detailed discussion on this topic, see, for example [40]. (As also noted by other authors, this analogy has been used to develop the contrast source inversion algorithm [38].)

### 2.2. Suppressing Undesired Scattering Effects

Based on the above discussion, it can be understood that the contrast sources induced in the OI can be considered as the *cause* for the scattered field data, similar to the current distribution of an antenna being the cause for its radiation pattern. More specifically, the scattered field at a given receiver due to a given transmitter can be thought as the weighted summation of all the contrast sources induced at different locations within the OI’s geometrical domain. (This is parallel to the concept of antenna arrays in which the field at a given location in space is the weighted summation of the effects of all the single antenna elements.) This can be better understood by observing the so-called *data equation* [41] which maps the contrast sources from the imaging domain *D* to the scattered field on the measurement domain *S* for the 2D scalar problem

(2) $$E^{\mathrm{scat}}\left(r\in S\right)=k_{b}^{2}\int_{D}g\left(r\in S,r^{\prime }\in D\right)w\left(r^{\prime }\in D\right)dr^{\prime }.$$

The above equation indicates that the scattered field at the receiver location r is affected by all the contrast sources weighted by the green’s function *g* of the background medium and the background wavenumber $k_{b}$. Therefore, one way to not *see* a specific part of the imaging domain, e.g., an undesired scatterer, is to ensure that the contrast sources induced in the undesired scatterer are small, and are ideally zero. Due to the fact that the dielectric profile within the imaging domain is unknown, we are not able to fully control the distribution of contrast sources within the imaging domain. However, since the contrast sources are affected by the incident NF distribution, $E^{\mathrm{inc}}$, through the so-called *domain equation* [41],

(3) $$w\left(r\in D\right)=\chi \left(r\in D\right)E^{\mathrm{inc}}\left(r\in D\right)+k_{b}^{2}\int_{D}g\left(r\in D,r^{\prime }\in D\right)w\left(r^{\prime }\in D\right)dr^{\prime },$$

we can use the incident NF distribution to *partially* control the distribution of the contrast sources.

**Figure 2:**
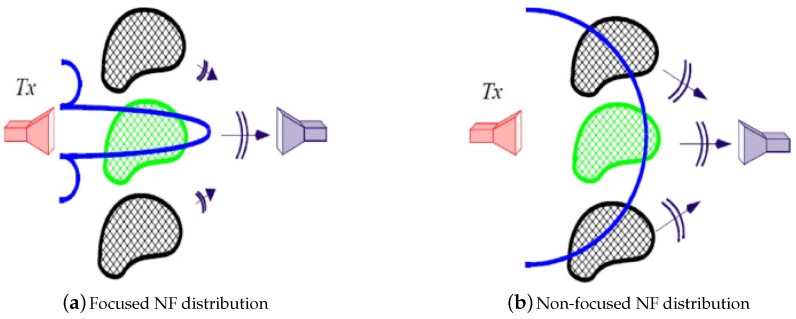
(**a**) represents the scenario in which a focused NF beam is used for irradiation of the OI (central green object); (**b**) represents the scenario in which a non-focused NF beam is used for irradiation. The side black objects are the undesired scatterers.

The idea to be pursued here has been demonstrated in Figure 2 where a focused incident NF in Figure 2a and a non-focused one in Figure 2b irradiate three distinct dielectric objects, with the central green one represents the OI, and the other two black ones represent the undesired scatterers. Due to the fact that the incident NF in Figure 2a is more focused toward the OI, the contrast sources within the undesired scatterers are more *likely* to be weaker in Figure 2a, and subsequently, the scattered data collected by the grey receiver in Figure 2a is more likely to contain less information about the undesired scatterers as compared to the grey receiver shown in Figure 2b. Therefore, if the main purpose is to see the OI (central object), Figure 2a offers an advantageous measurement scenario as the received signal is mainly due to the OI. We will investigate this further in Section 3 of this paper. (This is similar to our approach toward justifying an appropriate incident field for 2D transverse magnetic inversion [32].) Note that there exist situations in which focusing the incident field in one spot does not result in weaker contrast sources elsewhere; however, for situations where Born approximation is valid ($E\approx E^{\mathrm{inc}}$ thus $w=\chi E\approx \chi E^{\mathrm{inc}}$), focusing the incident field into a spotlight will weaken the contrast sources at other areas. That is why we have limited the scope of this paper to scenarios in which the total and incident fields share similar distributions.

It is also important to note that in some situations, the use of a focused incident field has a disadvantage compared to a non-focused one. In particular, if the transmitter in Figure 2 can vertically move up and down to scan the OI, then at some elevations the focused incident field will not irradiate the OI. On the other hand, the non-focused incident field might still be able to irradiate the OI due to its wider NF beam. This will result in the loss of some useful information for the focused incident field. One remedy for this situation is to steer the antenna toward the OI when we move the antenna vertically up and down to ensure that the focused beam always see the OI. (This is similar to the concept of stripmap and spotlight modes in synthetic aperture radars.)

### 2.3. Sufficient Measured Data

In the previous section, we discussed suppressing the effect of undesired scatterers to avoid including them as unknowns in the inversion algorithm. As noted earlier, if we decide to include undesired scatterers as part of the unknowns in the inversion process, we need to ensure that sufficient measured data are collected to reconstruct not only the OI but also the undesired scatterers. We now rely on Section 2.1 to further discuss this in an intuitive fashion. To this end, let us first note that the equivalent inverse source problems corresponding to the MWI problem (see Figure 1) are, in fact, antenna characterization problems which we often encounter in NF antenna measurement techniques. (In NF antenna measurement techniques, antenna characterization or diagnostics deals with finding the equivalent currents of the antenna under test from NF antenna measurements.) This is due to the fact that each set of these contrast sources can be thought as an unknown *antenna* radiating the measured scattered fields. From the theory of cylindrical and spherical NF antenna measurements [42], it is known that the number of required measured data points depends on both the wavelength of operation (λ) and the electrical size of the antenna. Noting the relation between the MWI and inverse source problems, it can be understood that the required sampling resolution for MWI will similarly depend on the size of the OI and the wavelength of operation. We also note that, in NF antenna measurements, the above criteria is for finding the fields *outside* the measurement domain (i.e., for performing NF to FF transformation), which is easier than reconstructing the fields interior to the measurement domain. For example, the required angular and vertical data sampling resolutions for a cylindrical NF antenna measurement system is [43]

(4) $$\Delta \varphi =\frac{\lambda }{2(a+\lambda )}\;\mathrm{and}\;\Delta z=\frac{\lambda }{2},$$

where *a* is the radius of the smallest cylinder that encloses the antenna under test. It is worthwhile to note that the above sampling resolution requires collecting two orthogonal field components at each measurement location, which are tangential to the measurement surface, i.e., $E_{\varphi }$ and $E_{z}$ in the cylindrical coordinates where the axis of the measurement cylinder is assumed to be on the *z*-axis. (In MWI, often one field component is collected at each measurement location, in most implementation $E_{z}$.)

Based on the above discussion and the similarity of the inverse scattering and inverse source problems, the parameter *a* in MWI can be thought as the smallest cylinder that encloses the OI and undesired scatterers. Therefore, it can be easily understood that the larger *a* with respect to the wavelength, the more measured data are necessary for performing successful inversion. (We re-emphasize that this approach is only being used to develop some intuitive understanding about the MWI data sampling resolution, and should not be considered as an exact governing formula for the MWI problem. For a more detailed discussion on retrievable information, see [44].) If we are not able to provide the inversion algorithm with sufficient information content (e.g., due to the physical size of the antennas or strong mutual coupling between them), an alternative option will be to reduce the number of unknowns, e.g., by *not* seeing some parts of the original imaging domain. Therefore, although 3D full-vectorial inverse scattering algorithms are the most accurate option for inverting the measured data as practical systems and objects are all 3D structures, it is still important to simplify the inversion process for the cases where the amount of measured data cannot support the retrieval of many unknowns associated with 3D full-vectorial inversion. For example, each *voxel* of a discretized imaging domain will have four complex unknowns in 3D full vectorial inversion: $E_{x}$, $E_{y}$, $E_{z}$, and the OI’s complex permittivity. Therefore, for a 100×100×100 discretized imaging domain, we will therefore have $4\times 10^{6}$ complex unknowns. (Regularization techniques are helpful in providing *vitual* data, e.g., enforcing smoothness by the Laplacian regularizer, that partially handle this huge number of unknowns.)

### 2.4. A Synthetic Test Case

**Figure 3:**
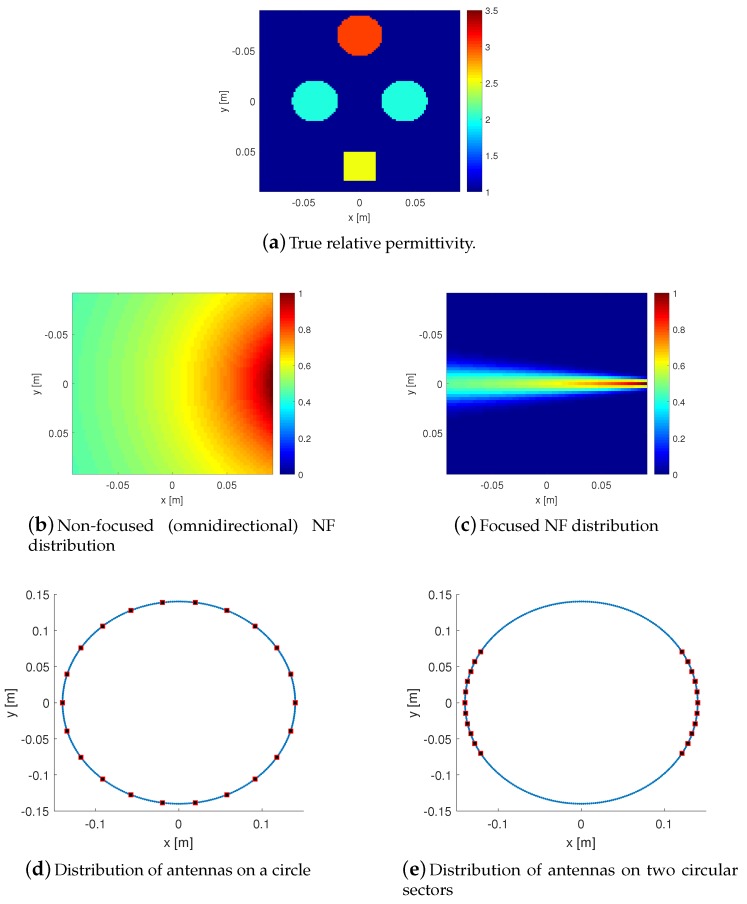
(**a**) represents the true relative permittivity profile. The two light blue circles are assumed to be the OI, and the other two side objects (red and yellow) are the undesired scatterers. (**b**,**c**) represent the non-focused (omnidirectional) and focused incident fields, respectively, which are used to irradiate the imaging domain. (**d**,**e**) represent two type of data collection schemes with antennas located either on a full circle or on a two circular sectors, respectively.

To demonstrate the concept above, we perform the following simulation study in which the utilized incident field is a numerical model, and does not represent an actual field. (In Section 3, we will focus on simulated fields due to practical NF antennas.) In this example, we consider a two-dimensional transverse magnetic MWI. The true dielectric profile considered is shown in Figure 3a which consists of four lossless scatterers in the xy plane. It is assumed that the two light blue circular scatters with the relative permittivity of 2.0 are the OI, and the other two (the yellow and red ones) with the relative permittivity of 2.5 and 3.0 are the undesired scatterers. The true profile was created on a $18\times 18\;{\mathrm{cm}}^{2}$ domain discretized into 100×100 square pulses. The frequency of operation is assumed to be 5 GHz. Two types of incident fields are considered to illuminate the imaging domain: the first one is an omnidirectional line source incident field, and the other is a focused incident field as shown in Figure 3b,c, respectively. The mathematical expression for the omnidirectional line source is $E^{\mathrm{inc}}=\hat{z}\frac{1}{4j}H_{0}^{2}(k_{b}|r-r^{\prime }\left|\right)$ where r represents the transmitter location, $r^{\prime }$ represents the observation point within the imaging domain, and $j^{2}=-1$. In addition, $H_{0}^{2}$ is the zeroth order Hankel function of the second kind. The focused incident field is then *modeled* (not, an actual field) by multiplying this omnidirectional incident field by $\cos^{m}\psi$ where ψ is the angle between the boresight axis of the antenna and the line connecting the antenna to the observation point in the imaging domain; e.g., see [27]. (The parameter *m*, which controls the focusing level, is set to be 300 here.) For all the antennas, the boresight axis is the line that connects the antenna to the centre of the imaging domain.

We consider two types of measurement domains: (1) a full circle and (2) a circular sector. In the first one, we place 22 antennas evenly distributed on a circle of 14 cm radius around the imaging domain as shown in Figure 3d. In the second scheme, we distribute these 22 antennas on two opposite $60^{\circ }$ sectors of the same measurement circle as shown in Figure 3e. Based on the analogy with the cylindrical NF antenna measurements noted in Section 2.3, the use of the circular sector measurement domain will not be as good as the full circular one; however, in some applications, we have to use this type of limited-view configurations as we may not have a full $360^{\circ }$ access around the imaging domain.

The irradiation of the imaging domain is performed using either the omnidirectional incident field or the focused one. In the irradiation process, when one antenna transmits, all the antennas including the transmit antenna collect the scattered field data. This scattered data is synthetically generated by a method of moment forward solver, and 3% noise is added to this data according to the formula shown in [45]. The inversion of the noisy scattered data is then performed by the multiplicative regularized Gauss–Newton inversion (MR-GNI) algorithm [16,19,46] on an imaging domain with the size of $18.4\times 18.4\;{\mathrm{cm}}^{2}$ discretized into 61×61 cells.

**Figure 4:**
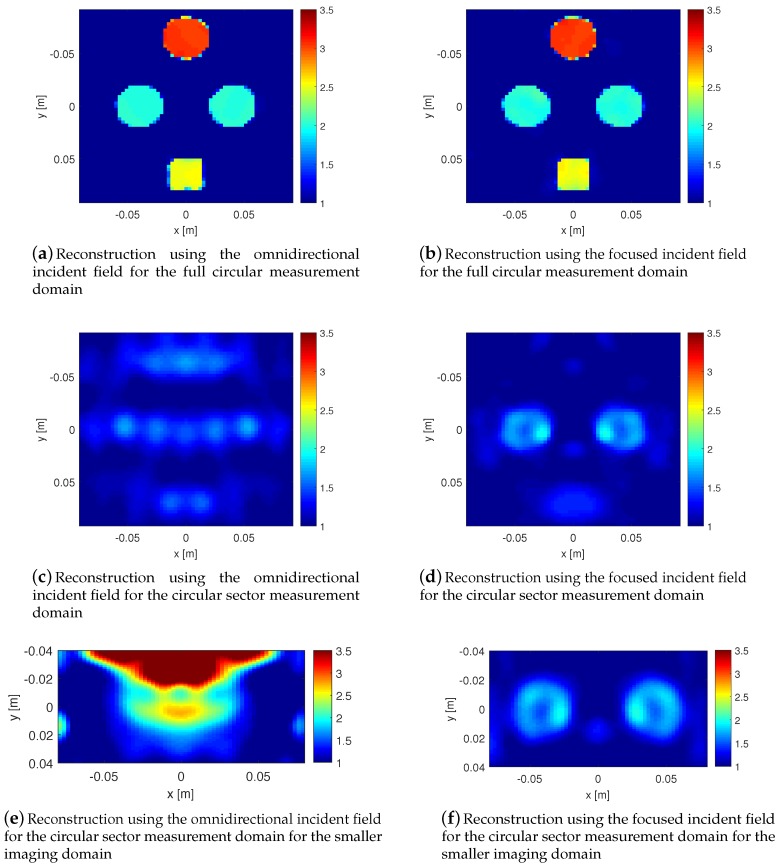
(**a**,**b**) represent the inversion of the data collected on the full circular measurement domain for two cases: omnidirectional and focused incident fields, respectively; (**c**,**d**) represent the inversion of the data collected on the circular sector measurement domain for two cases: omnidirectional and focused incident fields, respectively; (**e**,**f**) represent the inversion of the data collected on the circular sector measurement domain for two cases, omnidirectional and focused incident fields, respectively, with the smaller imaging domain that only includes the OI, and not the undesired scatterers.

The inversion results for the full circular measurement domain with the omnidirectional and focused incident fields are shown in Figure 4a,b, respectively. As can be seen, both images have good reconstruction accuracy. Next, we consider the circular sector measurement domain: its inversion results for the omnidirectional and focused incident fields are shown in Figure 4c,d, respectively. As can be seen, both images are poor due to suffering from limited view angles. However, it can be noticed that the use of the focused incident field enables us to at least see the OI (i.e., the two circular scatterers). This is due to the fact that the focused incident fields in this data collection configuration mainly see the OI, and not the undesired scatterers, and therefore, the measured scattered data on the circular sector measurement domain are mainly due to the OI. From a mathematical point of view, the use of the focused incident field has made the scattered field data less sensitive to the unknown undesired scatterers, and therefore the inversion algorithm can work with less informative data.

To investigate this further, we make the imaging domain smaller by shrinking it into a rectangular imaging domain of the size $16\times 8\;{\mathrm{cm}}^{2}$ discretized into 61×61 cells so as to only enclose the OI, and not the undesired scatterers. This will then completely ignore the presence of the undesired scatterers in the inversion algorithm. The inversion of the data collected on the circular sectors for this smaller imaging domain for the two incident field types are shown in Figure 4e,f. As can be seen, the inversion of the data due to the omnidirectional incident field is now even poorer since ignoring the presence of the undesired scatterers has resulted in significant modelling error in the inversion algorithm. However, as expected, the inversion of the data collected on the circular sectors due to the focused incident field still shows the overall geometry of the OI due to the data being less sensitive to the undesired scatterers. Finally, we note that, in this example, the beams of the focused incident fields are always toward the centre of the imaging domain (see the definition for $\cos^{m}\psi$ above). If, for example, the focused beams of the transmitting antennas were all parallel to the *x*-axis, some incident fields (i.e., those corresponding to the antennas at the top and bottom ends of the circular sectors) might not even interact with the OI, thus, reducing the information content of the data. (Of course, this is not an issue with omnidirectional incident fields.)

## 3. NF Beams and Results

The example presented in Section 2.4 utilized a numerically modelled incident field. In this section, we consider realistic incident NF distributions. To this end, we consider two antenna systems that are able to create NF focusing: (1) a NF plate which can create a subwavelength focused NF distribution on a line (line focusing) [47] and (2) a Bessel beam launcher which is able to generate spot focused NF distribution (spot focusing) and maintain it over a distance away from the launcher [48]. Herein, we use these two antennas to illuminate the OI so as to demonstrate the idea presented in the previous section. It should be noted that all the results presented herein have been simulated using ANSYS HFSS (Electronic Desktop 2018, ANSYS, Canonsburg, PA, USA).

### 3.1. Interrogation of Objects by the NF Plate

**Figure 5:**
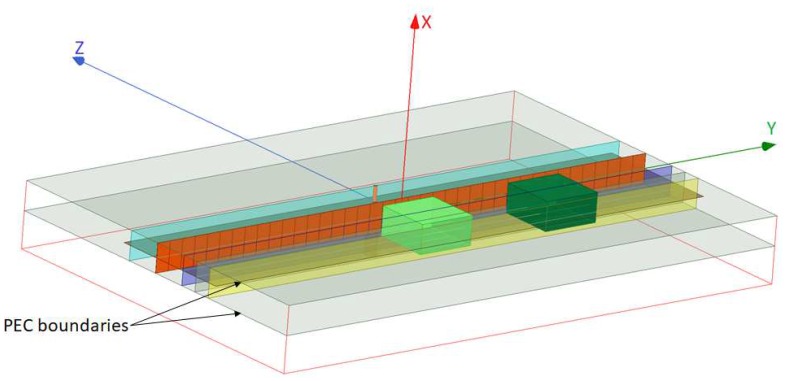
The simulated NF plate setup. The NF plate (orange plane), introduced in [47], is excited from the back by the inner conductor (orange cylinder) of a coaxial cable which lies on the excitation plane (light blue plane). This structure then illuminates the OI (light green dielectric box) and the undesired scatterer (dark green dielectric box). The dark blue and yellow planes show the focal plane and the receivers’ plane respectively. The two dielectric boxes have the same size of λ/20×λ/10×λ/10 along *x*-, *y*-, and *z*-directions, and have the same relative permittivity.

**Figure 6:**
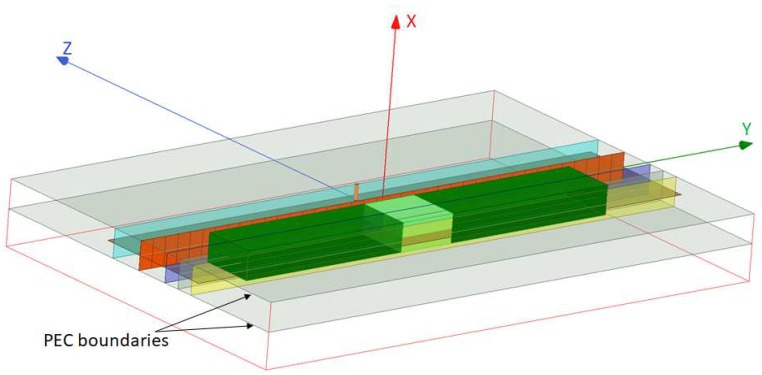
The simulated NF plate (orange plane) setup with a dielectric slab in front of it. The dielectric slab has the size of λ/20×2λ/3×λ/10. The description of the excitation and receiver planes are the same as Figure 5. The dielectric slab consists of an OI and two undesired scatterers. The OI is the light green box, and the two undesired scatterers attached to the OI are the dark green boxes.

The NF plate test setup, shown in Figure 5 and Figure 6, includes a thin passive plate with the length of about λ (orange plane) in the *y*-direction that consists of multiple capacitive elements and is placed within a parallel plate waveguide with the height of λ/20; see [47] for the details regarding this NF plate. At the frequency of 1 GHz, this passive NF plate is excited by the cylindrical waves emanating from the inner conductor of a coaxial cable that has been extended into the parallel plate waveguide. The excitation source is shown by an orange cylinder on the excitation plane (light blue plane) in Figure 5 and Figure 6. (The NF plate has the same separation of λ/15 from the excitation and focal planes.) Once illuminated by the impinging cylindrical waves, the NF plate creates a highly oscillating electromagnetic fields at the plate that subsequently results in a focused NF beam at the focal plane. As noted in [47], the NF plate is designed by back-propagating the desired focused NF distribution at the focal plane toward the NF plate to find the required field on the NF plate, then calculating the required surface impedance profile needed to support the existence of this field, and finally implementing this surface. Our simulated NF plate resulted in about λ/12 beamwidth on the focal plane, and was not a perfect *sinc* function compared to the one reported in [47], which also had a narrower beamwidth of λ/18. We also note that this NF plate can be regarded as an electromagnetic metasurface which transforms a given non-focused field into a desired focused one.

All of the simulation case studies for the NF plate setup are performed under one of the following two configurations: (i) *Configuration I* is the configuration in which the NF plate is present within the parallel plate waveguide to create a focused NF distribution, and (ii) *Configuration II* is the configuration that the NF plate is removed from the imaging setup in order to reduce the focusing of the NF distribution. (That is, Configuration II is obtained by removing the orange NF plate in Figure 5 and Figure 6.) For each of these two configurations, four different case studies are considered. The utilized objects (OI and the undesired scatterer) in all four case studies have the same relative permittivity of 1.50. In the first three case studies, the objects are dielectric boxes with the same dimensions of λ/20×λ/10×λ/10 along *x*-, *y*-, and *z*-directions, respectively, while in the fourth case study we elongate our dielectric box in the *y*-direction, thus creating a dielectric slab with the dimension of λ/20×2λ/3×λ/10 with the same relative permittivity of 1.50. In all these four cases, the separation between the closest facet of the dielectric objects to the NF plate is about λ/60. In addition, although our simulated configurations suffer from large $|S_{11}|$ values, these values are close to each other, thus making the comparison of the two configurations reasonable.

**Figure A1:**
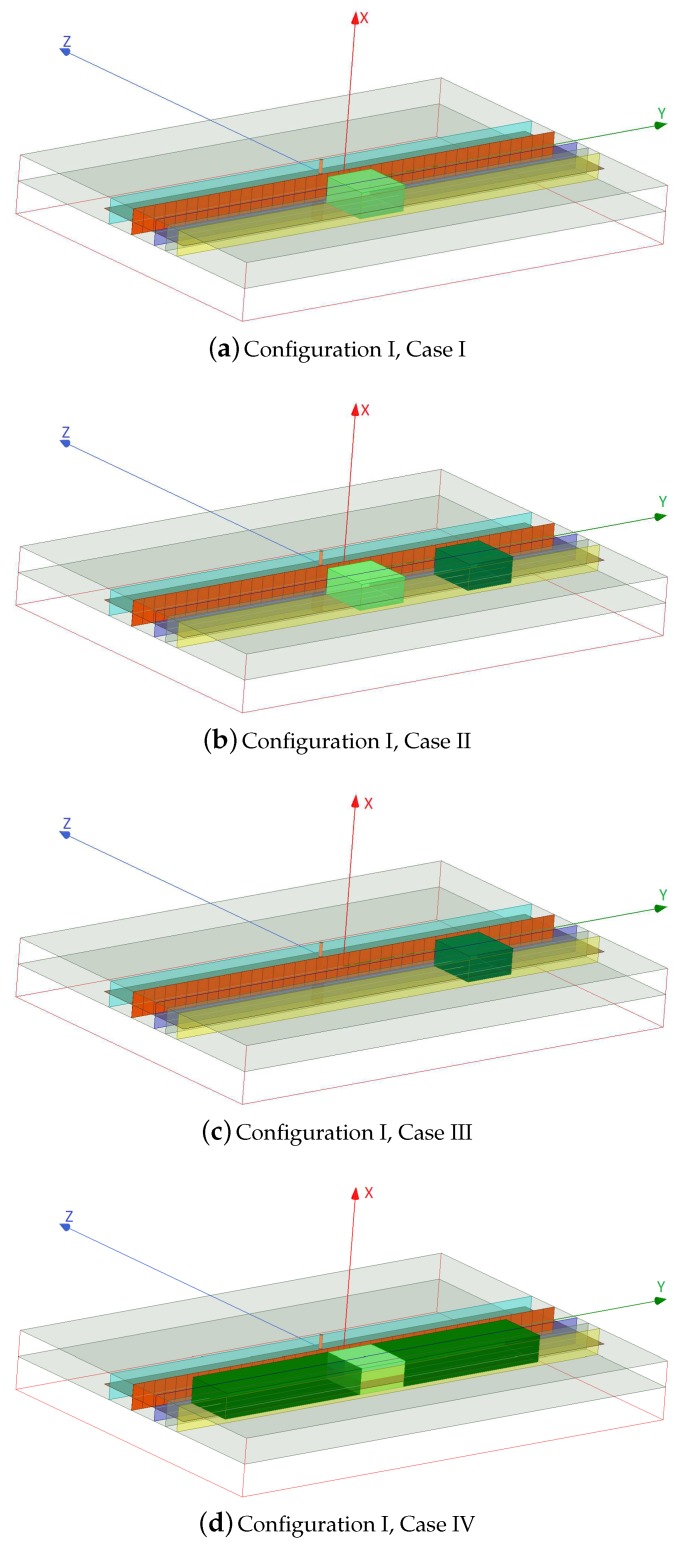
Summary of different case studies under Configuration I of the NF plate setup.

The four case studies are illustrated with figures in Appendix A. In Case I, as can be seen in Figure A1a in Appendix A, the only object present in the system is the OI that is placed exactly at the center with respect to the NF plate. In Case II (Figure 5), both the light and dark green dielectric boxes are present in the system. In both of these cases, the light green dielectric box represents the OI while the dark green dielectric box in Case II represents the undesired scatterer which is located λ/4 away from the center. Case III only consists of the undesired scatterer, as shown in Figure A1c. Finally, in Case IV (Figure 6), we have a dielectric slab that consists of an OI and two undesired scatterers. The OI, the light green box in Figure 6, has the same size and dielectric property as the OI in Cases I and II. Two undesired scatterers, the dark green boxes in Figure 6, have the same dielectric property as the OI. (The size of each of these undesired scatterers is λ/20×17λ/60×λ/10.)

Having defined our two configurations (i.e., presence and absence of the NF plate) and four case studies per configuration, we now irradiate these four different cases under each configuration, and then collect the resulting fields at the receivers’ locations. The receivers are located along the line of intersection between the yellow and brown planes in Figure 5 and Figure 6. This receiver line is parallel to the *y*-axis, and is separated from the NF plate by about λ/7.5. (Note that the receivers’ locations is not on the focal plane; the focal plane, which is λ/15 away from the NF plate, passes through the center of the objects.) The fields collected by the receivers in the presence of the objects are referred to as the total NF data. Subtracting the incident NF at the receivers’ locations from the total NF data, we find the *scattered NF data*. Analysis of the scattered NF data is the focus of the next section.

#### 3.1.1. Analysis of the Scattered NF Data

**Figure 7:**
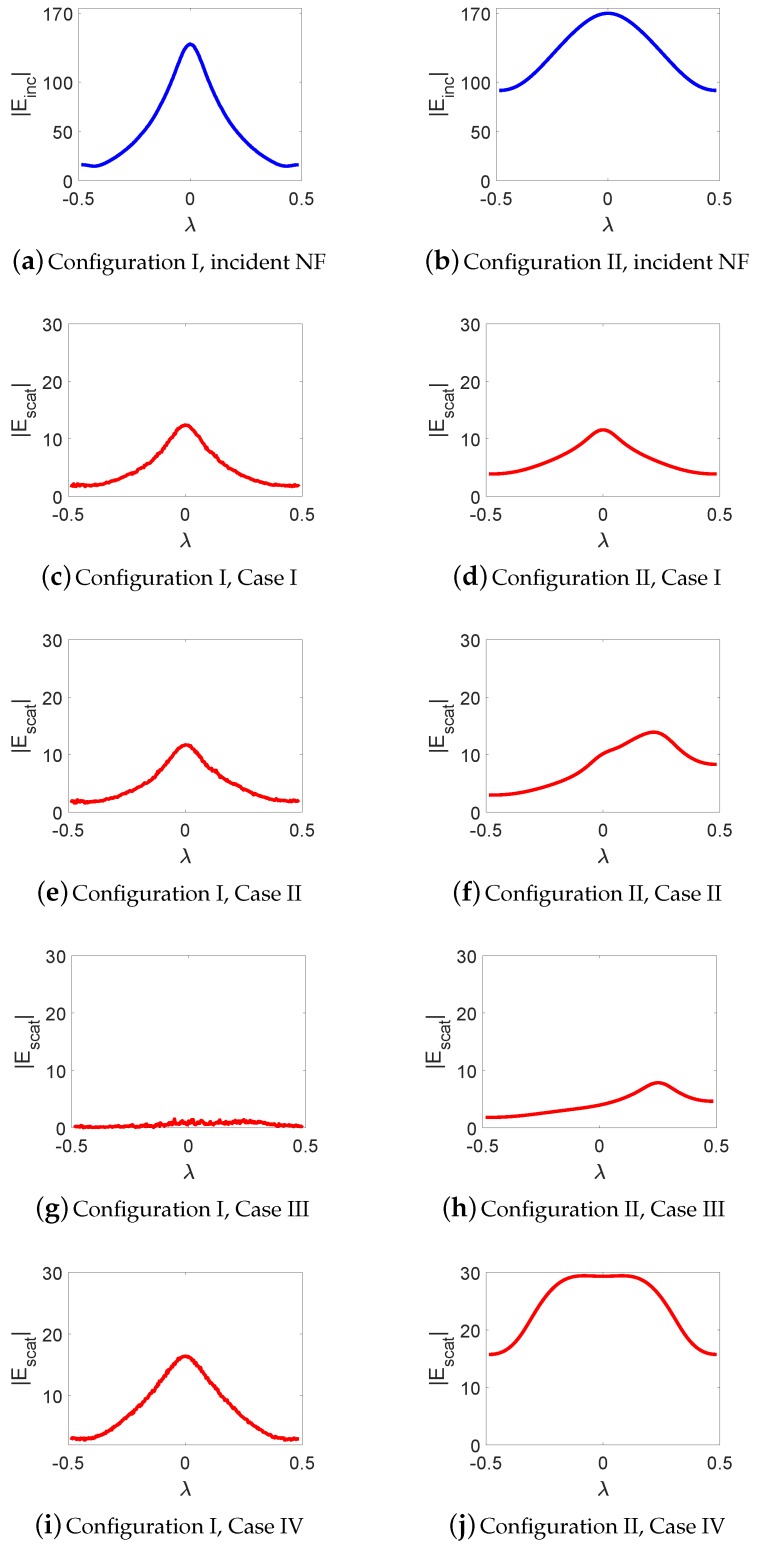
Configuration I represents the presence of the NF plate, and Configuration II represents its absence. (**a**,**b**) show the incident NF distributions at the receivers’ locations; (**c**–**j**) scattered NF data under the two configurations for Cases I to IV.

Herein, we discuss our observations from the comparison of the scattered NF data for the four case studies under both configurations. Prior to this, let us take a look at the magnitude of the incident NF distribution at the receivers’ locations for both configurations in Figure 7a,b which represent the presence and absence of the NF plate respectively. As can be seen, the presence of the NF plate has created a focused NF distribution. It should be noted that the focused incident field does not have the shape of the *sinc* function as expected based on the design presented in [47]. We speculate two reasons for this. First, the *sinc* behavior is to be expected at the focal plane whereas our receivers’ line is not on the focal plane. Second, our simulated structure can still be optimized; however, since our purpose is to study the performance of a more focused beam as compared to a non-focused one, the achieved incident field serves this purpose, which can be seen by comparing Figure 7a,b.

We now begin by comparing the scattered NF data for Cases I and II under our two configurations. Figure 7c,e show the scattered NF data for these two cases under Configuration I. As can be seen by comparing Figure 7c,e, the presence of the undesired scatterer has barely changed the scattered NF data. In other words, the imaging system employing this focused incident NF distribution is not very sensitive to the undesired scatterer. On the other hand, the scattered NF data for Configuration II (when the NF plate is removed) have been shown in Figure 7d,f: as can be seen, the effect of the undesired scatterer is now completely visible in the scattered NF data, and has shifted the maximum of the scattered NF data in Figure 7f. To further verify this, we consider Case III where only the undesired scatterer is present. The scattered NF data for this case under Configurations I and II have been shown in Figure 7g,h: as can be seen, the scattered NF data obtained in the presence of the NF plate (Configuration I) has much smaller magnitude compared to that obtained in the absence of the NF plate (Configuration II). In conclusion, the presence of the undesired scatterer is less “seen” by the receivers when the utilized irradiating source is the focused incident NF beam.

**Figure 8:**
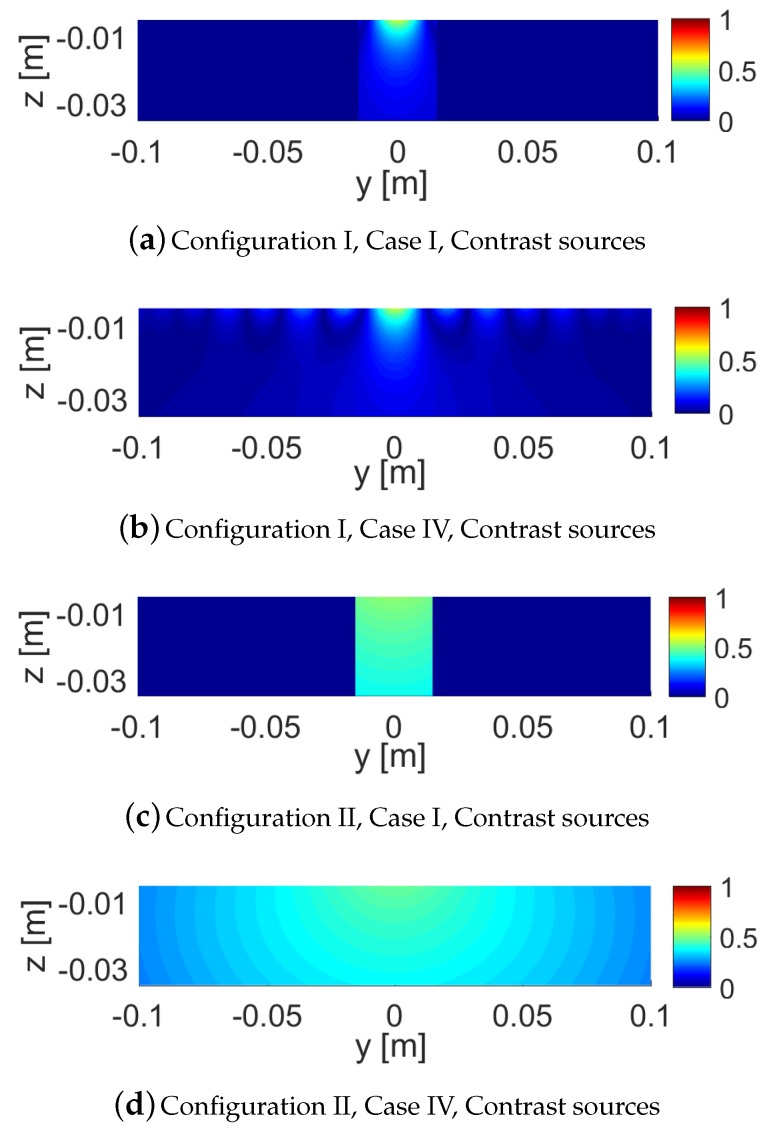
Induced contrast sources (their magnitudes) for Cases I and IV for both configurations: with the NF plate (Configuration I), and without the NF plate (Configuration II). The contrast sources in each configuration have been normalized with respect to the maximum of the incident field at that configuration.

As noted earlier, in Case IV, we elongate the light green dielectric box; see the new elongated light green dielectric box in Figure 6, which we refer to as the dielectric slab. The resulting scattered NF data for this dielectric slab have been shown in Figure 7i,j. Comparing Figure 7c,i for Configuration I and also comparing Figure 7d,j for Configuration II shows that elongating the dielectric box under Configuration I has not changed the resulting scattered NF data as much as that under Configuration II. In other words, the effects of elongating the OI is less visible with the NF plate present. To justify this, one may take a look at the induced contrast sources for Cases I and IV under both configurations as shown in Figure 8, and observe that the contrast sources associated with Cases I and IV undergo less changes in Configuration I compared to that in Configuration II. We speculate that this is important for 2D inversion (tomographic inversion) of 3D targets as signals arising from anywhere other than the cross section of interest contribute to the modelling error.

#### 3.1.2. Inversion Results

Now, let us consider inverting the scattered NF data collected using the NF plate setup. The choice of the inversion algorithm depends on many parameters including the number of measured NF data points. In MWI, it is often desirable to choose a nonlinear inversion algorithm (e.g., contrast source inversion or Gauss–Newton inversion methods) in which the OI’s dielectric profile is iteratively reconstructed. However, these nonlinear inversion algorithms are better suited when the OI is illuminated from different angles of incidence. (That is why we used a nonlinear inversion algorithm in Section 2.4.) As in Section 3.1.1, we have only illuminated the OI from one direction, the choice of nonlinear inversion algorithms is not appropriate. Therefore, we use a linear inversion algorithm in order to reconstruct the complex permittivity of the OI. In our linearized inversion approach, we assume that the scattered NF data is related to the unknown dielectric contrast χ linearly. That is, $E^{scat}=L\left(\chi \right)$ where L is a linear operator. As can be seen from Equation (2), this linear operator depends on both the green’s function of the system and the total field induced in the object. To determine L, we use a calibration object similar to the method presented in [7]. Herein, for simplicity, we reconstruct one relative complex permittivity value for the OI. In addition, we limit the data collection scheme to only one data point behind the centre of the OI on the receiver domain. Thus, the operator L simply becomes a complex number which is multiplied by the contrast to output the scattered NF value at the single measurement point. In other words, L becomes the slope of the line that linearizes the relation between the scattered NF data and the contrast around the contrast of the calibration object. Denoting the dielectric contrast of the calibration object as $\chi^{c}$, L simply becomes $E^{scat}\left(\chi^{c}\right)/\chi^{c}$ where $E^{scat}\left(\chi^{c}\right)$ is the scattered NF data due to the known calibration object. Once this L is found, the unknown contrast can be found as $\chi =E^{scat}/L$.

Now, consider the reconstruction of the permittivity value of the OI for the MWI setup shown in Figure 5 under our two configurations (with and without the NF plate) for case studies I and II. We consider the same dielectric boxes (with the size of λ/20×λ/10×λ/10) as presented earlier in Figure 5 as the OI and undesired scatterer, but now with a relative permittivity of 1.70, instead of 1.50. (As will be seen, the permittivity of 1.50 will be used as one of the calibration objects.) The inversion results for Configurations I and II for two different calibration objects (having the same size as the OI) are reported in Table 1. As can be seen, the inversion results for Cases I and II under the focused incident field illumination (Configuration I) do not change as much as those under Configuration II. (For example, compare the change from 1.90−j0.29 to 1.89−j0.32 with the stronger change from 1.74−j0.04 to 1.58−j0.25.) This demonstrates that Configuration I is less sensitive to the presence of the undesired scatterer. It should also be noted that the inversion result of Configuration II are more accurate. We speculate that this is due to the fact that the total fields in the calibration object and that in the OI are closer to each other for the non-focused incident beam, thus, making the utilized linear inversion algorithm more suitable for Configuration II.

#### 3.1.3. Separation Resolution Study

**Figure 9:**
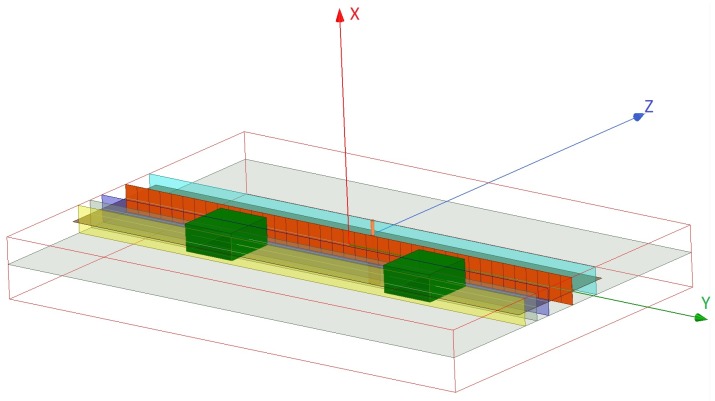
Separation resolution study. The simulated NF plate (orange plane) setup with two dielectric objects (dark green dielectric boxes) separated by about 0.13λ from each other. (In the figure, this separation has been *exaggerated* by a larger distance for illustration clarity.) The objects have the same size of λ/20×λ/10×λ/10 along the *x*-, *y*-, and *z*-directions, and have the same relative permittivity of 1.50. The description of the excitation and receiver planes are the same as Figure 5.

We now study the effect of using a focused incident NF distribution on the achievable separation resolution for one test case under the same two configurations described above. This case study for Configuration I is shown in Figure 9. As can be seen, there are two dielectric objects (the two dark green dielectric boxes in Figure 9) that have been placed approximately 0.13λ apart from each other in front of the NF plate. These two dielectric objects have the same relative permittivity of 1.50 and the same size of λ/20×λ/10×λ/10 in the *x*-, *y*-, and *z*-directions with the distance between their closest facet to the NF plate being about λ/60.

To perform this study, we assume that the NF plate moves along the *y*-direction to scan the two objects in the following three steps. Initially, its main beam is directly toward the first dielectric object, then the main beam is moved directly toward the gap between the two objects, and finally its main beam will be directly toward the second object. Similar to the previous section, this will constitute our Configuration I; for configuration II, we assume the same procedure but without the NF plate. In each of these three steps, the scattered NF data are collected on the receivers’ line residing on the yellow plane. For each of these steps, we have a set of distinct receivers which are mainly within the main beam of the NF plate. (The details of these steps and our simulation details are described in Appendix B.)

**Figure 10:**
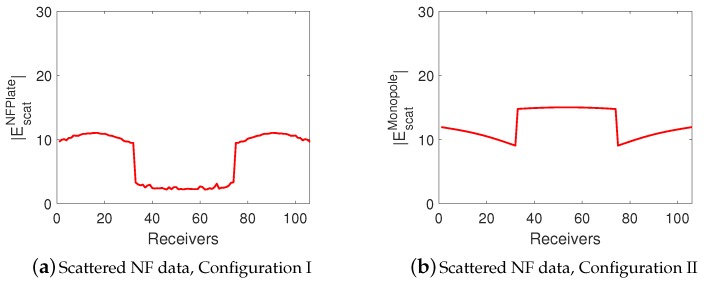
Separation resolution study. Scattered NF data for (**a**) Configuration I and (**b**) Configuration II.

We now discuss the obtained scattered NF data from this simulation study which have been depicted in Figure 10a,b. As can be seen from Figure 10a, the gap between the two dielectric boxes is detectable directly from the scattered NF data when the focused incident NF distribution is utilized. However, this is not the case when the NF plate is removed in Configuration II; this can be seen from Figure 10b. As can be seen in Figure 10a,b, the magnitude of the scattered NF data at the center of the figure, which is associated with the gap between the two dielectric objects, is *minimum* for Configuration I and *maximum* for Configuration II. Based on the concept of contrast sources that was explained earlier in Section 2.1, this can be explained as follows. In Configuration I, when the NF plate directly illuminates the gap, it will not induce significant contrast sources in the two dielectric objects. Therefore, the resulting scattered signal will be small. On the other hand, when the gap is illuminated in the absence of the NF plate (i.e., Configuration II), more contrast sources will be induced in the two dielectric objects due to the wider illuminating beam. Due to the symmetry of the objects, the induced contrast sources in the two objects will be in-phase. Therefore, the resulting two contrast sources can be thought as a two-element in-phase antenna array that subsequently generates a radiation peak toward its broadside ($-\hat{z}$ direction in Figure 10). This, therefore, justifies why the maximum of the scattered NF data in Configuration II occurs at the center of the Figure 10b. (The central receiver corresponds to the broadside direction.)

### 3.2. Interrogation of Objects by the Bessel Beam Launcher

**Figure 11:**
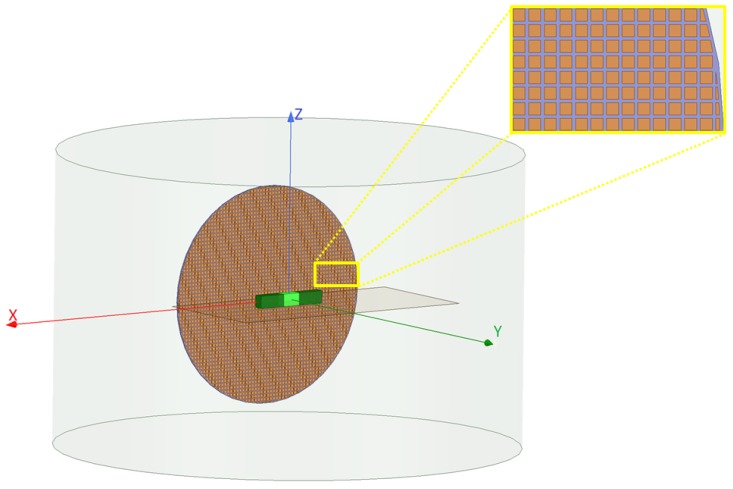
The simulated Bessel beam launcher setup. The Bessel beam launcher (orange circular structure), introduced in [48], is parallel to the *xz* plane. A more detailed view of the capacitive sheet is shown in the inset. The brown plane (*xy* plane) contains the cross section of three dielectric boxes, all of which have the same relative permittivity of two. This figure represents our Case II study where the light green dielectric box, with the size of λ/2×λ/4×λ/3, represents the OI, and the two dark green dielectric boxes, with the size of 3λ/4×λ/4×λ/3, serve as the undesired scatterers.

**Figure 12:**
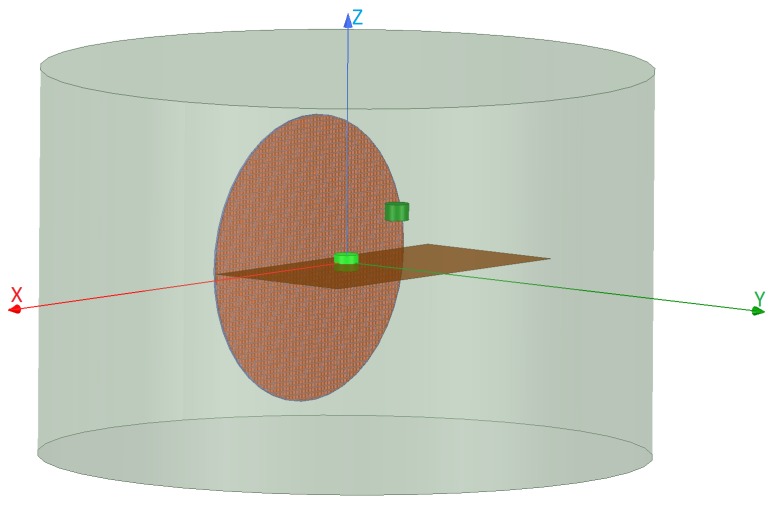
The simulated Bessel beam launcher setup. This represents Case IV where the light green circular cylinder (with the radius of λ/4 and the height of λ/3) represents the OI and the dark green circular cylinder, with the same size and dielectric properties as the OI, represents the undesired scatterer.

In the previous section, the idea of suppressing undesired scattering effects for MWI was studied using the NF plate. One limitation of this study lies in the fact that the OI and the undesired scatterer(s) are located on one line. This was due to the fact that the utilized NF plate provides 1D focusing. To consider more general cases, we have simulated the Bessel beam launcher presented in [48,49] by the use of ANSYS HFSS so as to apply it to MWI applications. This Bessel beam launcher, shown in orange in Figure 11 and Figure 12, is a planar structure capable of focusing electromagnetic fields into a 2D spot in front of the launcher and then maintains this focused beam over some distance away from the launcher. This distance is referred to as the non-diffracting zone of the launcher, after which the focused beam starts to diffract [48]. (The non-diffractive zone of this launcher is about 2.1λ [49].) This launcher utilizes a leaky radial waveguide in order to generate the Bessel beam [48]. This waveguide consists of a capacitive impedance sheet located over a ground plane (separated by 1 mm) which is fed by a coaxial cable. This capacitive impedance sheet is made of square patch elements (with the length of less than λ/10 printed on both sides of a substrate with $\epsilon_{r}=6.15$ and the height of h=0.127 mm. The diameter of this launcher is about 6λ and its frequency of operation is 10 GHz [49]. The simulated Bessel beam launcher is capable of generating electric field whose normal component, the $\hat{y}$ direction in Figure 11 and Figure 12, is a truncated zeroth-order Bessel function of the first kind, and has a first-null beamwidth of less than λ. That is, theoretically, the incident field in the $\hat{y}$-direction will be in the form of [48]

(5) $$E_{y}=J_{0}\left(k_{xz}\sqrt{x^{2}+z^{2}}\right)e^{-jk_{y}y},$$

where $J_{0}$ is the zeroth-order Bessel function of the first kind, and $k_{xz}^{2}+k_{y}^{2}=k_{b}^{2}$ where $k_{xz}$ is the transverse wavenumber, $k_{y}$ is the normal wavenumber, and $k_{b}$ is the wavenumber in the background medium (free space in our case).

**Figure A3:**
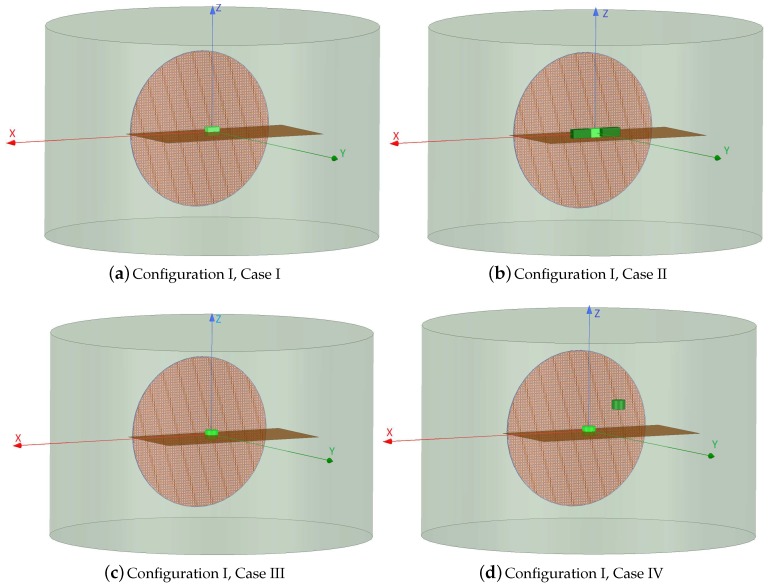
Summary of different case studies under Configuration I of the Bessel beam launcher setup.

**Figure A4:**
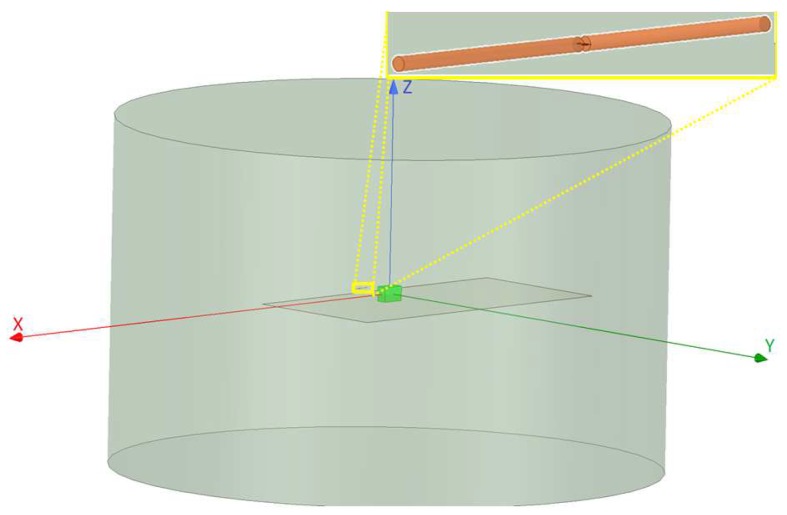
Configuration II in which a horizontal dipole antenna replaces the Bessel beam launcher for Case I.

Similar to the NF plate case, we consider two configurations. Configuration I uses the Bessel beam launcher to irradiate the objects. On the other hand, in Configuration II, a dipole antenna replaces the Bessel beam launcher to serve as our non-focused incident beam (see Figure A4 in Appendix A). For each of these configurations, we consider four case studies. In Case I, a rectangular dielectric box with the relative permittivity of two and the size of λ/2×λ/4×λ/3 is placed at the center of the launcher and about λ away from the launcher (similarly, about λ away from the dipole antenna in Configuration II) as shown in Figure A3a. Case II not only considers the light green box as the OI, but also includes the two undesired scatterers, the dark green boxes in Figure 11, attached to the OI. These undesired scatterers have the same relative permittivity as the OI, each of which with the size of 3λ/4×λ/4×λ/3. Case III, see Figure A3c, includes a new OI that is a circular dielectric cylinder with the relative permittivity of four, height of λ/3, and the radius of λ/4 located about λ away from the center of the Bessel beam launcher. Finally, Case IV adds this dark green dielectric cylinder as an undesired scatterer to Case III. Figure 12 shows Case IV where the light and dark green cylinders represent the OI and undesired scatterer, respectively. In Case IV, the OI and undesired scatterer have the same dielectric property, and the undesired scatterer is offset by (λ,λ/2,λ) with respect to the center of the OI. (The center of the OI is the origin of the coordinates.)

Finally, it should be noted that all the simulations concerning Configuration II (i.e., in the absence of the Bessel beam launcher) have been preformed assuming that the dipole is horizontally oriented along the *x*-axis. Since neither a horizontal dipole nor a vertical one can generate a dominant $E_{y}$ component (similar to the Bessel beam launcher), we have oriented the dipole along the *x*-axis so that a dominant $E_{x}$ can be achieved in Configuration II. This enables us to compare two different incident fields (focus and non-focused ones) with their dominant electric field components lying in the imaging plane (xy plane), similar to the transverse electric microwave tomography.

#### 3.2.1. Analysis of the Scattered NF Data

**Figure 13:**
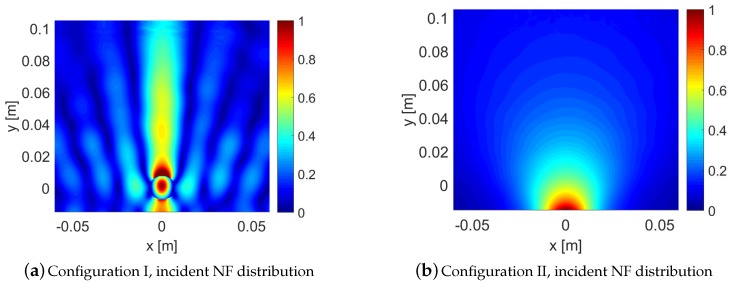
Normalized magnitude of the incident NF distribution for (**a**) Configuration I (Bessel beam) and (**b**) Configuration II (dipole).

**Figure 14:**
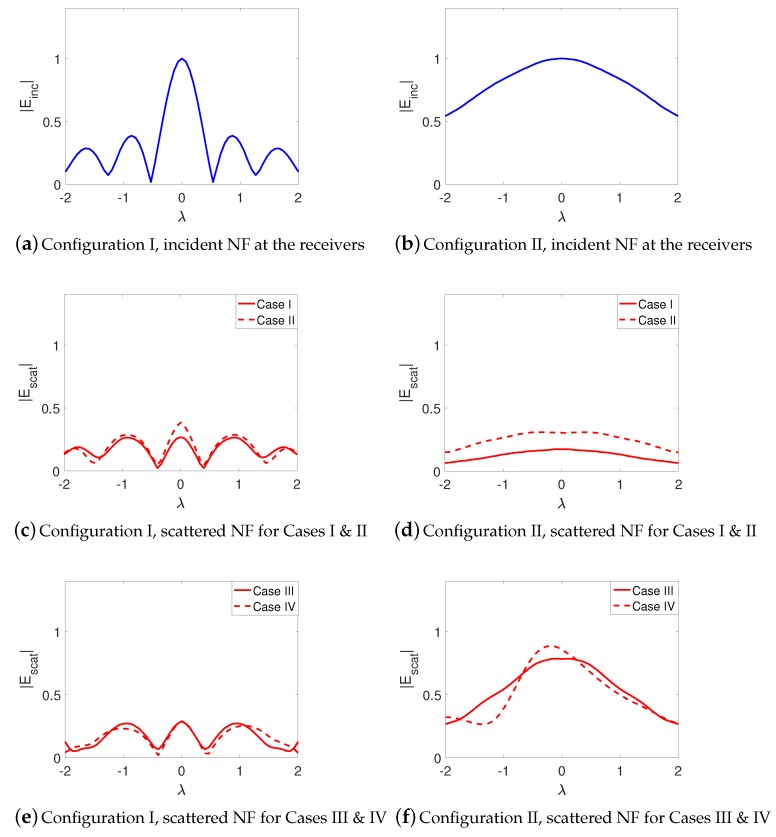
(**a**,**b**) normalized magnitude of the incident NF at the receiver line; (**c**–**f**) scattered NF at the receiver line for Configurations I and II regarding Case I (single small box), Case II (elongated box consisting of an OI and two undesired scatterers), Case III, (single small cylinder) and Case IV (small cylinder with an offset extra undesired scatterer) respectively. In Configuration I, these fields correspond to the $\hat{y}$ component of the electric field, and, in Configuration II, they correspond to the $\hat{x}$ component of the electric field.

**Figure 15:**
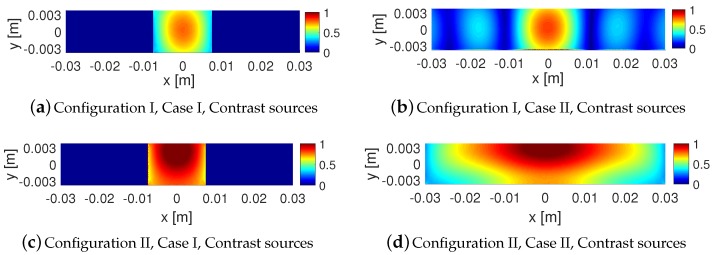
Induced contrast sources (their magnitudes) for Cases I and II for both configurations: using the Bessel beam launcher (Configuration I), and using the dipole antenna (Configuration II). The contrast sources in each configuration have been normalized with respect to the maximum of the incident field at that configuration.

The incident NF beams within the xy plane for Configurations I and II are shown in Figure 13a,b. In addition, the measurement domain (receivers’ locations) is taken to be a line in the xy plane and parallel to the *x*-axis, which is 2λ away from the OI’s center. Over this line, we have distributed 61 receivers that are evenly spaced. The incident NFs on the measurement domain have also been plotted in Figure 14a,b. (Note that the first-null beamwidth of the Bessel beam in Figure 14a is about λ.) The scattered NF data (i.e., the scattered fields at the receivers’ locations) for Cases I to IV for both configurations are shown in Figure 14c,f. As can be seen in these figures, the change in the scattered NF data due to the introduction of the undesired scatterer is less in Configuration I than that in Configuration II. Based on the discussion presented in Section 2.2, we speculate that this is due to the fact that the induced contrast sources have undergone less changes in Configuration I when the undesired scatterers were introduced. This has been demonstrated in Figure 15 where the contrast sources for both configurations regarding Cases I and II have been plotted: as can be seen, the contrast sources in Case I and II are more similar under Configuration I. (In addition, note that the contrast sources w outside the box in Case I for both configurations are exactly zero since the contrast χ outside the box is zero.)

#### 3.2.2. Inversion Results

A simple linear inversion algorithm, the same as the one used in Section 3.1.2, is employed to invert the scattered NF data. To this end, we utilize a known calibration object with a complex relative permittivity value close to that of the OI. Using this calibration object, we linearize the nonlinear inverse scattering problem, and then reconstruct the complex permittivity of the OI using one measured data point located at (0,2λ,0), which is directly behind the OI.

Now, let us first consider the inversion results for Cases I and II under our two configurations. As listed in Table 2, the calibration object has a relative permittivity of 1.50 and the size of λ/2×λ/4×λ/3 (same size as the OI in Cases I and II). The achieved reconstructed value (real and imaginary parts) under Configuration I shows less changes compared to those under Configuration II when we go from Case I to Case II. However, as can be seen the inversion results under Configuration I are quantitatively less accurate. The same observation holds when comparing the reconstructed values for Cases III and IV under the two configurations, see Table 2. That is, under Configuration I, when we go from Case III to Case IV, the reconstructed value shows smaller changes. However, quantitatively, Configuration II yields more accurate results.

The observation that the reconstructed values under Configuration I undergo less changes when the undesired scatterers are introduced indicates that Configuration I has provided less sensitivity to the presence of these undesired scatterers. However, it is important to discuss why the reconstructed permittivity under Configuration I is less accurate. Herein, we present a few speculations to justify this. First, the linearized inversion approach assumes that the total field in the calibration object and the OI are identical. We speculate that this assumption is more accurate for the dipole antenna due to having a broader and more uniform beam. (Note that a given inversion algorithm can be more suitable to a given measurement setup depending on its assumptions.) Second, the Bessel beam has the so-called “self-healing” property [50,51], which could reduce the sensitivity of the data to the unknown permittivity. Third, the polarization of the Bessel beam launcher used for imaging is $\hat{y}$ and that of the dipole is $\hat{x}$. Therefore, the induced contrast sources in the OI can be thought as some dipole antennas oriented in the $\hat{y}$- and $\hat{x}$-directions for Configurations I and II, respectively. The measured data point, located at (0,2λ,0), will then be mainly along the axes of these dipoles in Configuration I and along the broadside of these dipoles in Configuration II. This could also result in less sensitivity for Configuration I.

## 4. Discussion and Conclusions

Broadly speaking, this paper proposes that tailoring the contrast sources induced in the objects can be used advantageously in MWI. This approach has the potential to suppress (or enhance) the sensitivity of the measured data with respect to specific regions of the imaging domain, e.g., a region which requires more accurate assessment. It was noted that we do not have full control over tailoring contrast sources since the induced total fields within the objects are dependent on the objects’ unknown dielectric properties. Due to this difficulty, we have limited the scope of this paper to scenarios in which the induced total field distributions inside homogeneous objects are similar to the illuminating incident field distributions. For the cases where this (Born) assumption does not hold, one option is to utilize an estimate of the dielectric profile of the objects (which might be obtained via a different imaging modality), and then use this prior knowledge to create an incident field distribution that results in a desired total field distribution in the objects.

We also noted the importance of tailoring the incident NF beam of the irradiating antenna, as opposed to its FF pattern, for NF MWI. In this paper, we have investigated two NF beams for imaging which satisfy our desired property: a focused NF distribution. We envision that the area of electromagnetic metasurfaces will offer a systematic tool to synthesize appropriate incident NF distributions to be utilized for imaging (e.g., see [52]). This is due to the fact that metasurfaces can transform a given excitation electromagnetic field to a desired one by imposing appropriate surface boundary conditions [53]. In addition, in this work, we did not consider tailoring the polarization of the incident field for the benefit of imaging, which can also be pursued in future.

In this paper, we have only considered focused NF beams with the purpose of minimizing the sensitivity of the collected scattering data to undesired scatterers. However, depending on the application area and purpose, one can employ incident NF beams of different distributions to optimize the retrieval of various features of interest. In addition, within the scope of our assumptions, we have intuitively discussed that this focused NF beam approach can limit the geometrical support of the induced contrast sources, and can therefore result in less required measured data points as compared to scenarios in which wider NF beams are utilized. In addition, one of the other potential advantages of focused NF beams, which has not been investigated here, is the ability to enhance the SNR of the measured data. In some applications, such as those which operate in lossy medium, enhancement of the SNR is critical to enhance the achievable reconstruction accuracy and resolution.

In summary, this paper demonstrated that the use of focused NF beams can suppress some undesired scattering effects. From a broader point of view, the main message of this paper is the suggestion that the incident NF beam, as an MWI system design component, can be advantageously utilized to optimize some imaging aspects, and not just to irradiate the objects.

## Figures and Tables

**Table 1:** The reconstructed OI’s permittivity for Cases I and II under the two configurations.

Cases	Calibration Object’s Permittivity	Configuration I (Focused)	Configuration II (Non-Focused)
Case I	1.20	1.90−j0.29	1.74−j0.04
Case II	1.20	1.89−j0.32	1.58−j0.25
Case I	1.50	1.65−j0.03	1.71−j0.01
Case II	1.50	1.66−j0.01	1.57−j0.23

**Table 2:** The inversion results for Cases I to IV under the two configurations: with the Bessel beam (Configuration I) and the dipole antenna (Configuration II).

Cases	Calibration Object’s Permittivity	Configuration I (Focused)	Configuration II (Non-Focused)
Case I	1.50	1.51−j0.11	2.00−j0.13
Case II	1.50	1.61−j0.01	2.16−j1.30
Case III	3.50	3.52−j0.16	3.58−j0.63
Case IV	3.50	3.54−j0.18	3.77−j0.88

**Figure A2:**
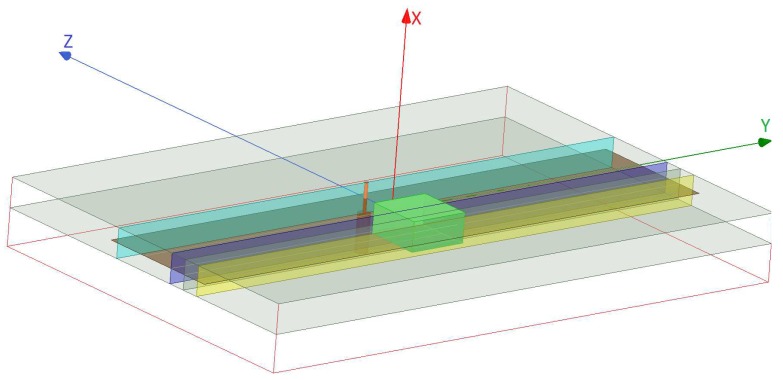
Configuration II, which is in the absence of the NF plate, for Case I.

## Appendix A. Visual Summary of the Different Test Cases

In this Appendix, we show additional figures to better demonstrate the studied test cases in Section 3. This illustration starts by the test cases under Configuration I considered in Section 3.1 as shown in Figure A1a–d. As noted in the paper, Configuration II for Section 3.1 is simply achieved by merely removing the NF plate. Due to this, only one case (Case I) under Configuration II is illustrated herein; see Figure A2. Next, the test cases considered in Section 3.2 are shown in Figure A3a–d. Configuration II for these test cases are simply achieved by replacing the Bessel beam launcher with a dipole antenna. Due to this, only one test case (Case I) under Configuration II is shown herein; see Figure A4.

## Appendix B. Simulation Details of the Separation Resolution Study

In this Appendix, we elaborate on how the separation resolution simulation study (see Section 3.1.3) was carried out by the use of ANSYS HFSS. To this end, we have placed two dielectric objects with the separation of about 0.13λ from each other in the NF plate setup under Configurations I and II. Due to the simulation setup, it was not possible to keep the two dielectric objects stationary, and then move the NF plate to scan the objects in the three steps as described in Section 3.1.3. To handle this, instead of moving the NF plate and keeping the objects stationary, we do the reverse procedure: the two separated objects are moved together parallel to the NF plate in three steps. In Step I, the first dielectric object is located exactly at the center and in front of the NF plate. In Step II, the gap between the two objects is located at the center with respect to the NF plate, and finally in Step III the second dielectric object is placed at the center with respect to the NF plate. The two separated objects at each of these three steps are interrogated, and then the resulting scattered NF data are collected at the receivers’ line (located on the yellow plane in Figure 9).

Now, we will explain how the scattered NF data have been collected in each of the steps above. In this study, we have placed the total number of 106 receiving points along the line of intersection between the yellow and brown planes in Figure 9. This line, which we refer to as the receivers’ line, is parallel to the *y*-axis and has been separated by about λ/7.5 from the NF plate. At each of the three steps above, only those receivers that reside exactly behind the dielectric object being illuminated (Steps I and III) or the gap being illuminated (Step II) are used for data collection. (In other words, the receiving points used in each step are located mainly within the main beam of the NF plate.) We then plot these three sets of the scattered NF data together to create Figure 10a for Configuration I and Figure 10b for Configuration II. Finally, it should be mentioned that, due to the symmetry of the NF plate setup as well as the symmetric arrangement of the two dielectric objects, we have obtained the scattered NF data in Step III by flipping the scattered NF data from Step I.

## References

[1] Pastorino M. (2010). Microwave Imaging.

[2] Bucci O.M., Bellizzi G., Borgia A., Costanzo S., Crocco L., Massa G.D., Scapaticci R. (2017). Experimental Framework for Magnetic Nanoparticles Enhanced Breast Cancer Microwave Imaging. IEEE Access.

[3] Fear E.C., Hagness S.C., Meaney P.M., Okoniewski M., Stuchly M.A. (2002). Enhancing breast tumor detection with near-field imaging. IEEE Microw. Mag..

[4] Oliveri G., Salucci M., Anselmi N., Massa A. (2017). Compressive Sensing as Applied to Inverse Problems for Imaging: Theory, Applications, Current Trends, and Open Challenges. IEEE Antennas Propag. Mag..

[5] Persson M., Fhager A., Trefna H.D., Yu Y., McKelvey T., Pegenius G., Karlsson J.E., Elam M. (2014). Microwave-Based Stroke Diagnosis Making Global Prehospital Thrombolytic Treatment Possible. IEEE Trans. Biomed. Eng..

[6] Mobashsher A.T., Abbosh A.M., Wang Y. (2014). Microwave System to Detect Traumatic Brain Injuries Using Compact Unidirectional Antenna and Wideband Transceiver with Verification on Realistic Head Phantom. IEEE Trans. Microw. Theory Tech..

[7] Nikolova N.K. (2017). Introduction to Microwave Imaging.

[8] Wu Z., Wang H. (2017). Microwave Tomography for Industrial Process Imaging: Example Applications and Experimental Results. IEEE Antennas Propag. Mag..

[9] Gibbins D., Byrne D., Henriksson T., Monsalve B., Craddock I.J. (2017). Less Becomes More for Microwave Imaging: Design and Validation of an Ultrawide-Band Measurement Array. IEEE Antennas Propag. Mag..

[10] Hopfer M., Planas R., Hamidipour A., Henriksson T., Semenov S. (2017). Electromagnetic Tomography for Detection, Differentiation, and Monitoring of Brain Stroke: A Virtual Data and Human Head Phantom Study. IEEE Antennas Propag. Mag..

[11] Boverman G., Davis C., Geimer S., Meaney P.M. (2017). Image Registration for Microwave Tomography of the Breast Using Priors from Non-Simultaneous Previous Magnetic Resonance Images. IEEE J. Electromagn. RF Microw. Med. Biol..

[12] Mojabi P., LoVetri J. (2016). Composite Tissue-Type and Probability Image for Ultrasound and Microwave Tomography. IEEE J. Multiscale Multiphys. Comput. Tech..

[13] Jiang H., Li C., Pearlstone D., Fajardo L. (2005). Ultrasound-guided microwave imaging of breast cancer: Tissue phantom and pilot clinical experiments. Med. Phys..

[14] Scapaticci R., Bellizzi G., Catapano I., Crocco L., Bucci O.M. (2014). An Effective Procedure for MNP-Enhanced Breast Cancer Microwave Imaging. IEEE Trans. Biomed. Eng..

[15] Firoozy N., Komarov A.S., Landy J., Barber D.G., Mojabi P., Scharien R.K. (2015). Inversion-Based Sensitivity Analysis of Snow-Covered Sea Ice Electromagnetic Profiles. IEEE J. Sel. Top. Appl. Earth Obs. Remote Sens..

[16] Ostadrahimi M., Mojabi P., Zakaria A., LoVetri J., Shafai L. (2013). Enhancement of Gauss–Newton Inversion Method for Biological Tissue Imaging. IEEE Trans. Microw. Theory Tech..

[17] Grzegorczyk T.M., Meaney P.M., Kaufman P.A., diFlorio Alexander R.M., Paulsen K.D. (2012). Fast 3D Tomographic Microwave Imaging for Breast Cancer Detection. IEEE Trans. Med. Imaging.

[18] De Zaeytijd J., Franchois A., Eyraud C., Geffrin J.M. (2007). Full-Wave Three-Dimensional Microwave Imaging with a Regularized Gauss Newton Method; Theory and Experiment. Antennas Propag. IEEE Trans..

[19] Abubakar A., Habashy T.M., Pan G., Li M.K. (2012). Application of the Multiplicative Regularized Gauss Newton Algorithm for Three-Dimensional Microwave Imaging. IEEE Trans. Antennas Propag..

[20] Palmeri R., Bevacqua M.T., Crocco L., Isernia T., Donato L.D. (2017). Microwave Imaging via Distorted Iterated Virtual Experiments. IEEE Trans. Antennas Propag..

[21] Mojabi P., LoVetri J. (2009). Overview and Classification of Some Regularization Techniques for the Gauss-Newton Inversion Method Applied to Inverse Scattering Problems. IEEE Trans. Antennas Propag..

[22] Golnabi A.H., Meaney P.M., Paulsen K.D. (2013). Tomographic Microwave Imaging with Incorporated Prior Spatial Information. IEEE Trans. Microw. Theory Tech..

[23] Neira L.M., Veen B.D.V., Hagness S.C. (2017). High-Resolution Microwave Breast Imaging using a 3-D Inverse Scattering Algorithm with a Variable-Strength Spatial Prior Constraint. IEEE Trans. Antennas Propag..

[24] Baran A., Kurrant D., Zakaria A., Fear E., LoVetri J. Breast cancer imaging using microwave tomography with radar-derived prior information. Proceedings of the 2014 USNC-URSI Radio Science Meeting (Joint with AP-S Symposium).

[25] Mojabi P., LoVetri J., Shafai L. (2011). A Multiplicative Regularized Gauss-Newton Inversion for Shape and Location Reconstruction. IEEE Trans. Antennas Propag..

[26] Meaney P.M., Navin K., Yagnamurthy K.D.P. (2002). Pre-scaled two-parameter Gauss-Newton image reconstruction to reduce property recovery imbalance. Phys. Med. Biol..

[27] Bayat N., Mojabi P. (2016). A Mathematical Framework to Analyze the Achievable Resolution from Microwave Tomography. IEEE Trans. Antennas Propag..

[28] Li D., Meaney P.M., Raynolds T., Pendergrass S.A., Fanning M.W., Paulsen K.D. (2004). Parallel-detection microwave spectroscopy system for breast imaging. Rev. Sci. Instrum..

[29] Ostadrahimi M., Mojabi P., Gilmore C., Zakaria A., Noghanian S., Pistorius S., LoVetri J. (2011). Analysis of Incident Field Modeling and Incident/Scattered Field Calibration Techniques in Microwave Tomography. IEEE Antennas Wirel. Propag. Lett..

[30] Meaney P., Paulsen K., Chang J., Fanning M., Hartov A. (1999). Nonactive antenna compensation for fixed-array microwave imaging. II. Imaging results. IEEE Trans. Med. Imaging.

[31] Bourqui J., Okoniewski M., Fear E.C. (2010). Balanced Antipodal Vivaldi Antenna with Dielectric Director for Near-Field Microwave Imaging. IEEE Trans. Antennas Propag..

[32] Bayat N., Mojabi P. (2015). On an Antenna Design for 2D Scalar Near-Field Microwave Tomography. Appl. Comput. Electromagn. Soc. J..

[33] Bayat N., Mojabi P. (2014). The Effect of Antenna Incident Field Distribution on Microwave Tomography Reconstruction. Prog. Electromagn. Res..

[34] Bayat N., Mojabi P. Near-field microwave imaging using focused near-field beams: An approach to mitigate undesired scattering effects. Proceedings of the 2nd URSI Atlantic Radio Science Meeting (URSI AT-RASC).

[35] Bellizzi G.G., Iero D.A.M., Crocco L., Isernia T. (2018). Three-Dimensional Field Intensity Shaping: The Scalar Case. IEEE Antennas Wirel. Propag. Lett..

[36] Bellizzi G.G., Bevacqua M.T., Battaglia G.M., Crocco L., Isernia T. Advances in Target Conformal SAR Deposition For Hyperthermia Treatment Planning. Proceedings of the 2nd URSI Atlantic Radio Science Meeting (URSI AT-RASC).

[37] Crocco L., Donato L.D., Iero D.A.M., Isernia T. (2012). A New Strategy to Constrained Focusing in Unknown Scenarios. IEEE Antennas Wirel. Propag. Lett..

[38] Van den Berg P.M., Kleinman R.E. (1997). A contrast source inversion method. Inverse Probl..

[39] Oristaglio M., Blok H. (1995). Wavefield Imaging and Inversion in Electromagnetics and Acoustics.

[40] Chew W.C., Wang Y.M., Otto G., Lesselier D., Bolomey J.C. (1994). On the inverse source method of solving inverse scattering problems. Inverse Probl..

[41] Abubakar A., van den Berg P.M., Mallorqui J.J. (2002). Imaging of biomedical data using a multiplicative regularized contrast source inversion method. IEEE Trans. Microw. Theory Tech..

[42] Parini C., Gregson S., McCormick J., van Rensburg D.J. (2014). Theory and Practice of Modern Antenna Range Measurements.

[43] Balanis C. (2005). Antenna Theory: Analysis and Design.

[44] Bucci O.M., Isernia T. (1997). Electromagnetic inverse scattering: Retrievable information and measurement strategies. Radio Sci..

[45] Abubakar A., van den Berg P.M., Semenov S.Y. (2004). A Robust iterative method for Born inversion. IEEE Trans. Geosci. Remote Sens..

[46] Mojabi P., LoVetri J. (2009). Microwave Biomedical Imaging Using the Multiplicative Regularized Gauss-Newton Inversion. IEEE Antennas Wirel. Propag. Lett..

[47] Grbic A., Jiang L., Merlin R. (2008). Near-field Plates: Subdiffraction focusing with patterned surfaces. Science.

[48] Ettorre M., Grbic A. (2012). Generation of Propagating Bessel Beams Using Leaky-Wave Modes. IEEE Trans. Antennas Propag..

[49] Ettorre M., Rudolph S.M., Grbic A. (2012). Generation of Propagating Bessel Beams Using Leaky-Wave Modes: Experimental Validation. IEEE Trans. Antennas Propag..

[50] Bouchal Z., Wagner J., Chlup M. (1998). Self-reconstruction of a distorted nondiffracting beam. Opt. Commun..

[51] Ahmed N., Lavery M.P.J., Huang H., Xie G., Ren Y., Yan Y., Willner A.E. Experimental demonstration of obstruction-tolerant free-space transmission of two 50-Gbaud QPSK data channels using Bessel beams carrying orbital angular momentum. Proceedings of the 2014 European Conference on Optical Communication (ECOC).

[52] Zvolensky T., Gollub J.N., Marks D.L., Smith D.R. (2017). Design and Analysis of a W-Band Metasurface-Based Computational Imaging System. IEEE Access.

[53] Epstein A., Eleftheriades G.V. (2016). Huygens’ metasurfaces via the equivalence principle: Design and applications. J. Opt. Soc. Am. B.

